# Transforming cardiology with AI: the eko CORE 500 digital stethoscope

**DOI:** 10.1007/s12471-025-01952-5

**Published:** 2025-03-26

**Authors:** Pim van der Harst, Hendrik Nathoe

**Affiliations:** Department of Cardiology, Division of Heart and Lungs, University Medical Centre Utrecht, Utrecht, The Netherlands

**Keywords:** Artificial intelligence, Cardiovascular disease, Digital stethoscope

## Transforming cardiology with AI

Artificial intelligence is increasingly integrated into cardiovascular medicine, with new technologies entering clinical practice. This section provides a brief evaluation of recently available AI-driven products, reflecting the authors’ personal perspectives on their utility and limitations. The views expressed do not constitute an endorsement by the NVVC.

## Introduction

The stethoscope, one of the most recognisable medical instruments, was invented in 1816 by René Laennec, who first used a rolled-up sheet of paper to amplify chest sounds. Over time, this tool has evolved, but its basic function has remained largely unchanged. With the development of digital stethoscopes and AI-assisted diagnostics, the landscape of auscultation and bedside cardiovascular assessment is changing. A recently developed digital stethoscope integrates additional features, including AI-driven capabilities [1, 2]. One of the authors purchased this stethoscope at the AHA Congress last year and installed the accompanying software in the United States. However, the device is not yet available on the EU market, and its accompanying software may not be accessible through EU app stores. Here, we describe our first experience with this stethoscope at the University Medical Centre Utrecht.

## Technical features and capabilities

**Fig. 1:**
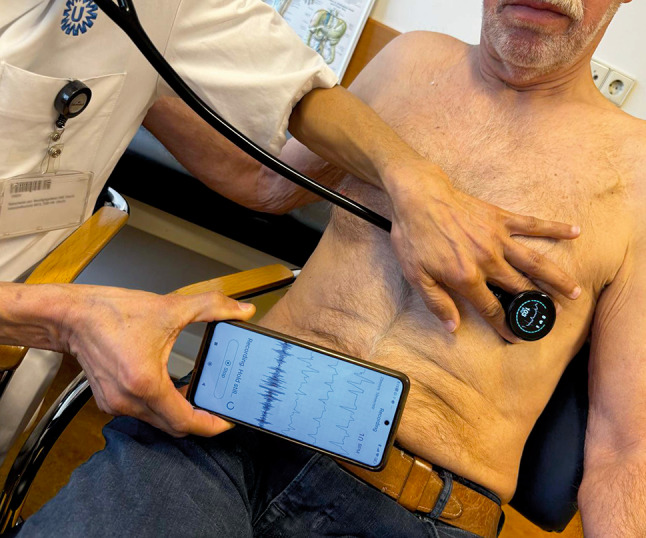
An image of the digital stethoscope in use, displaying heart rate directly on the device and ECG/sound recordings on a Bluetooth-connected mobile phone

- *ECG recording*: three electrodes positioned around the diaphragm allow simultaneous 3‑lead ECG recording. The ECG is instantaneously displayed on the stethoscope (Fig. 1). The ECG recordings and heart sounds can be captured by the software on Bluetooth-connected iOS/Android device. The quality of the ECG recording was sufficient to detect atrial fibrillation (AF) in our experience.
- *Sound*: 40 × amplification and active noise cancellation, according to the manufacturer. Based on our personal experience, it indeed helps to detect fainter heart sounds more easily and may be of value in nosier settings. The recording function is also of interest for teaching. However, further research is needed to determine whether it will impact clinical care and outcomes.
- *AI for analysis of murmur and AF*: unfortunately, the AI-powered murmur and AF detection are only available via subscription. The device integrates with the SENSORA platform of the manufacturer for hospital-based screening, enhancing murmur classification and LVEF detection, with potential efficiency benefits in screening for heart failure and valvular disease. Testing these features was not possible for us due to the absence of a subscription.

## Potential clinical applications

Given our initial experience, we believe that this device might be useful and probably should be studied in following settings.

- *Emergency departments and triage settings*: In overburdened hospitals, where echocardiographers are understaffed, this device could serve as a pre-screening tool, helping to prioritise patients for ECG and echocardiographic assessment.
- *General practice and primary care*: Many general practitioners lack the expertise or equipment for advanced cardiac diagnostics. The AI-assisted auscultation and ECG functionality of this stethoscope could help identify patients needing referral, reducing unnecessary cardiology visits while ensuring early detection of significant pathology.
- *Rural and remote medicine*: In low-resource settings or regions with few cardiologists—such as parts of Africa, South America, and Southeast Asia—this stethoscope could facilitate early detection of valvular disease and arrhythmia, potentially reducing the burden of advanced stage cardiac disease.
- *Humanitarian and field medicine*: Its portability, AI support, and ECG recording capabilities make it an interesting tool for mobile clinics, refugee health services, and disaster response teams. It enables basic cardiac assessments in areas with limited access to advanced imaging.
- *Telemedicine and remote consultation*: With data-sharing options, this device could support virtual cardiology consultations, allowing primary care providers in remote locations to share recordings with specialists for second opinions.

## Clinical considerations

- *Validation*: Although the advantages of the additional features on this stethoscope seem interesting, the added value for clinical care and outcomes as well as its cost-utility has not been well studied.
- *Regulatory and accessibility*: The lack of regulatory clearance beyond FDA 510(k) and certification prevents the device from being widely available outside the United States.
- *AI functionality: *While marketed as having AI-powered murmur detection, this feature requires an additional paid subscription to either Eko + (for individuals) or the SENSORA platform (for hospital-based screening and diagnostics). Limited data have been published on the value of detecting murmurs or low ejection fraction more easily or at an earlier stage.
- *Competing technologies*: As (consumer-based) ECG recording by patients and clinical handheld echocardiography devices become more accessible and affordable, clinicians must consider balancing the benefits of AI-enhanced auscultation versus direct cardiac imaging.

## Future perspectives

The integration of digital stethoscopes with ECG recording and AI-powered analysis represents a promising advancement in cardiovascular diagnostics. However, widespread adoption will depend on regulatory approvals, seamless integration with local electronic health records, and robust clinical validation. While AI-assisted auscultation and sound amplification show potential in early studies, their impact on clinical decision-making, patient outcomes, and cost-effectiveness has yet to be established. Further research is needed to assess their comparative utility relative to the traditional stethoscope and handheld echocardiography in early detection and triage, particularly in resource-limited, emergency, and primary care settings.
